# Solitary Necrotic Nodule of the Liver: A Benign Mimicker of Malignancy

**DOI:** 10.7759/cureus.52835

**Published:** 2024-01-23

**Authors:** Nadir Miry, Younesse Najioui, Anass Haloui, Nassira Karich, Amal Bennani

**Affiliations:** 1 Department of Pathology, Faculty of Medicine and Pharmacy, Oujda, MAR

**Keywords:** benign liver pathology, liver benign nodule, liver nodule, solitary necrotic nodule, liver pathology

## Abstract

Solitary necrotic nodule (SNN) of the liver is an uncommon and benign finding in liver pathology. Typically, it appears as a single and asymptomatic lesion, primarily located at the subcapsular region of the right lobe of the liver. Unfortunately, it is easy to mistake this benign lesion for a primary or secondary neoplastic lesion, making it a potential diagnosis pitfall for liver malignancies. The diagnosis of SNN can be difficult to determine as the imaging findings frequently lack specificity. This brings out the importance of histomorphological examination to accurately identify this lesion, and to rule out any possible malignancies.

We report here the case of a 35-year-old woman with a history of squamous cell carcinoma of the cervix, who presented a solitary nodule on her liver that was falsely diagnosed as a metastatic lesion in the liver at imagery. The aim of this article is to highlight the importance of using special stains and immunohistochemical staining for diagnosing SNN and excluding any necrotic metastases of the liver. We demonstrated that the absence of a reticulin meshwork in the necrotic core should prompt consideration of a necrotic metastasis in the liver, rather than a solitary necrotic nodule.

## Introduction

Solitary necrotic nodule (SNN) of the liver is a rare and indolent lesion, usually incidentally discovered in liver parenchyma, first described by Shepherd et al. [1]. It is an asymptomatic lesion, slightly more common in males compared to females [2]. From a radiological perspective, it can be challenging to distinguish SNN from primary and secondary liver tumors, particularly in patients with history of malignancy or existing malignancies, due to their similar imaging characteristics [3]. Therefore, clinical and radiological aspects alone may not be sufficient to establish a conclusive diagnosis. Histological examination remains the most reliable approach to confirm such diagnosis; it shows, microscopically, a central necrotic core with preserved reticulin network, surrounded by a relatively thick and fibrous capsule [4]. When SNNs coexist with a malignant tumor, distinguishing them could be extremely difficult. Therefore, a complete resection of the nodule with close follow-up may be considered as the best treatment option for SNN [5].

## Case presentation

**Figure 1:**
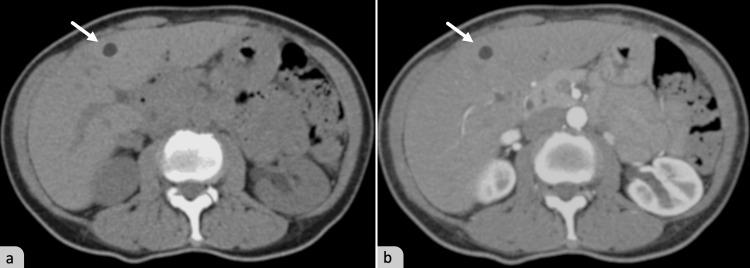
Abdominal axial CT scan showing a hypointense lesion (arrow) in segment IVb of the right liver (a), the lesion does not show enhancement after contrast injection (b).

We present here the case of a unique solitary necrotic nodule of the liver mimicking a hepatic metastasis, in a 35-year-old female patient with a previous history of squamous cell carcinoma of the cervix. The lesion was discovered incidentally during a follow-up investigation. Abdominal CT scan revealed a solitary and hypodense lesion in segment IVb of the liver, approximately 22mm in size, with no enhancement after contrast injection (Figure 1). Given the patient’s history of malignancy, the nodule was highly suggestive of a metastatic lesion, leading to an initial diagnosis of a metastatic lesion in the liver. The patient underwent a surgical resection of the nodule.

**Figure 2:**
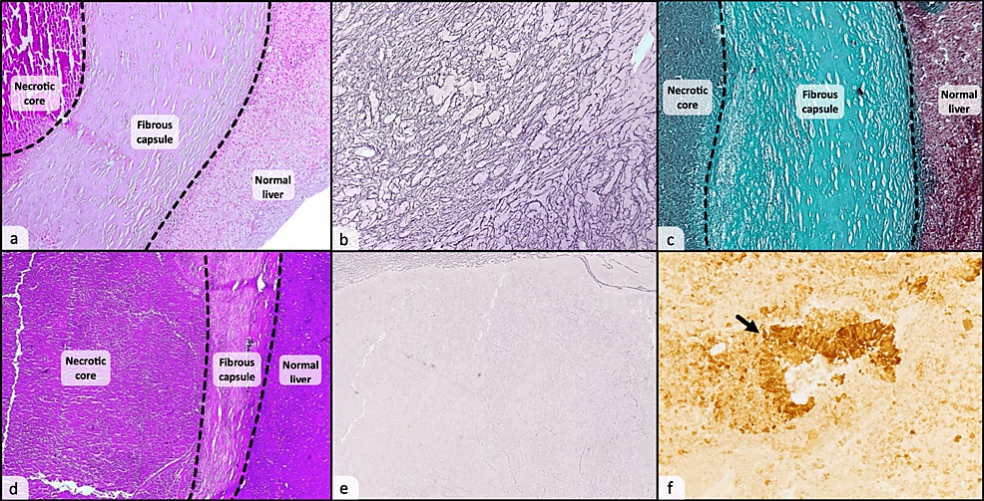
Photomicrographs of the solitary necrotic nodule (first patient) show a central necrotic core surrounded by a peripheral thick and fibrous capsule (a: H&E, x40). Silver stain reveals a well preserved reticulin meshwork in the necrotic core (b). Trichrome stain demonstrates the fibrous nature of the peripheral capsule (c). The necrotic metastasis (second patient) shows the same morphological characteristics as a solitary necrotic nodule (d: H&E, x40). The reticulin meshwork is completely absent in the necrotic center, demonstrated by a negative silver stain (e). Cytokeratin immunostaining reveals residual and partially necrotic glands in the background, confirming the metastatic nature of the lesion (f). H&E: Hematoxylin and Eosin Stain

Histological examination of the specimen showed a nodular lesion located in the subcapsular region of the liver, the central zone consisted of completely necrotic tissue, with a thick and well-demarcated ring of fibrous tissue bordering the central core. The adjacent liver tissue was found to be normal without any signs of malignancy (Figure 2A). Further analysis of the lesion using silver stain revealed a well-preserved reticulin network within the necrotic area (Figure 2B), while Trichrome stain confirmed the fibrous nature of the surrounding capsule (Figure 2C). No micro-organisms were found on both periodic acid-Schiff (PAS) and Ziehl stains. Based on these findings, the lesion was diagnosed as a solitary necrotic nodule of the liver.

To highlight the importance of immunostaining, we added the case of a solitary nodule of the liver in a patient with a colorectal carcinoma, the histomorphological examination of the lesion revealed the same morphological pattern as shown in the first case, with a completely necrotic nodule, surrounded by a fibrous capsule, with no viable tumor cells or glands (Figure 2D), silver nitrate stain was negative in the necrotic core, and it did not show the characteristic reticulin meshwork of the normal liver parenchyma (Figure 2E); the use of a cytokeratin (CK) immunostain clearly highlighted some abnormal and partially necrotic glands (Figure 2F). Based on these findings, the liver nodule in the second case was diagnosed as a necrotic metastasis of the liver, of probable colorectal origin.

Therefore, we strongly recommend a complete histomorphological assessment of any necrotic nodule in the liver, using special stains (nitrate silver stain) along with immunohistochemical markers, to rule out a metastatic lesion of the liver, especially when the reticulin meshwork is disrupted or absent.

## Discussion

SNN of the liver is a rare and benign finding that can mimic primary or secondary liver malignancies [4]. Only histomorphological examination could establish a definitive diagnosis. Typically, it is a small and well-demarcated round or oval lesion measuring less than 3cm in size [2]. Although usually solitary, cases of multiple SNNs have been previously reported [6]. It is mostly found in the subcapsular region of the right lobe of the liver [2]. The pathogenesis of such a lesion is still unclear, although it may be associated with parasitic infection, trauma, or regression from sclerosing liver hemangiomas [3,7].

The lesion is usually discovered incidentally during radiological examinations in the majority of cases. Radiologically the lesion appears nodular and well-circumscribed, showing overlapping characteristics with metastatic lesions in the liver [2]. CT scan shows a homogenous, hypodense lesion. MR imaging shows a hypointense signal on T1-weighted images and a hyperintense signal on T2-weighted images, with or without intralesional septations [5]. Usually there is no contrast enhancement on CT or MR imaging, which can be helpful in distinguishing a solitary necrotic nodule from a hepatic metastasis [4,5]. However, cases with peripheral rim-like enhancement have been described [7].

Histologically, the nodule is composed of a central necrotic core that may contain eosinophils, calcifications, cholesterol, foamy macrophages, and inflammatory cells. The necrotic core is surrounded by a fibrous capsule admixed with inflammatory cells [3,8]. Silver nitrate stain can be useful in differentiating SNN from a necrotic metastasis in the liver by demonstrating an intact reticulin meshwork in the necrotic core, in case the of a SNN. An immunohistochemical stain may also be used in order to reveal any partially preserved tumor cells or glandular structures that could be inconspicuous on H&E stain.

The immunoreactivity of necrotic tissue remains unpredictable and is directly related to the integrity of targeted antigens in the necrotic tissue [9]. The use of immunohistochemical markers on completely necrotic tissue has been scarcely described in the literature. However, some studies aimed to determine the sensitivity and specificity of widely used antibodies. A study conducted by Judkins et al. evaluated the utility of CK, CD45 and S100 on necrotic tumors. They concluded that CK (EA1/EA3) has the highest rates of sensitivity and specificity among other markers, with no reported false-positive cases. In contrast, CD45 and S100 should be cautiously interpreted in necrotic tissue [10]. Another study by Bogajewska-Rylko et al. demonstrated that immunohistochemical stains should be considered a reliable tool to demonstrate the epithelial nature of a necrotic metastasis [11]. 

In our case, differentiating SNN from a necrotic metastasis of the liver was crucial to confirm any relapse linked to the patient’s history of squamous cell carcinoma of the cervix.

## Conclusions

Solitary necrotic nodule of the liver is a benign lesion with close radiological similarities to neoplastic lesions of the liver. Distinguishing SNN from other neoplastic lesions of the liver based solely on imaging characteristics could be very challenging. Histomorphological examination of the lesion remains the gold standard for establishing the correct diagnosis. The aim of this case report is to highlight the importance of special stains and immunohistochemical markers to rule out possible necrotic metastasis in the liver and to confirm the diagnosis of solitary necrotic nodule.
